# Calf muscle pump tensing as a novel maneuver to improve the diagnostic performance of detecting patent foramen ovale during transesophageal echocardiography

**DOI:** 10.3389/fneur.2023.1116764

**Published:** 2023-01-25

**Authors:** Jianbo Zhu, Anni Chen, Lei Zhu, Yun Li, Yunyi Tang, Yanhua Huang, Hualiang Shen, Zhenzhen Jiang, Xiatian Liu

**Affiliations:** Department of Ultrasound, Shaoxing People's Hospital, Shaoxing, China

**Keywords:** patent foramen ovale, transesophageal echocardiography, calf muscle pump, provocative maneuver, Valsalva maneuver

## Abstract

**Objective:**

The Valsalva maneuver is the most sensitive provocative maneuver for patent foramen ovale detection. However, nearly half of patients are unable to perform the Valsalva maneuver well. The aim of this study was to investigate the mechanism of action of calf muscle pump tensing (TENSE) as a novel patent foramen ovale (PFO) provocative maneuver and to evaluate the diagnostic value for PFO and the effect on right-to-left shunt volume compared with the Valsalva maneuver.

**Methods:**

This study prospectively investigated 171 patients who were highly suspected to have PFO clinically. Five patients with atrial septal defects newly diagnosed on transesophageal echocardiography (TEE) were excluded. 166 patients were injected with agitated saline under three provocative maneuvers: Valsalva maneuver, TENSE, and Valsalva + TENSE combined maneuver. The patients were divided into the effective Valsalva group (*n* = 93) and ineffective Valsalva group (*n* = 73) according to whether they could perform an effective Valsalva maneuver. TENSE consisted of the straightening of both lower limbs, and when the right atrium was filled with microbubbles, the patient performed instantaneous ankle dorsiflexion movements while maintaining dorsiflexion for 3–5 s.

**Results:**

Overall, the PFO detection rate of the Valsalva + TENSE combined maneuver (78 [50.1%]) was significantly higher than that of the Valsalva maneuver (51 [30.7%]) and TENSE maneuver (57 [34.3%]) (*P* < 0.001). In the patients who were able to perform an effective Valsalva maneuver, the PFO detection rate by TENSE was not significantly different from that by the Valsalva maneuver (Valsalva 37/93 [39.8%] vs. TENSE 31/93 [33.3%], *P* > 0.05), while for the patients who performed an ineffective Valsalva maneuver, the PFO detection rate by the TENSE maneuver was higher than that by the Valsalva maneuver (TENSE 26/73 [35.6%] vs. Valsalva14/73[19.2%], *P* = 0.017).

**Conclusion:**

TENSE is a simple and effective provocative maneuver in the diagnosis of PFO using TEE and can assist the Valsalva maneuver. For patients who cannot perform an effective Valsalva maneuver, TENSE can be an alternative to the Valsalva maneuver to some extent.

## 1. Introduction

Patent foramen ovale (PFO) is defined as the failure of the septum primum to completely fuse with the septum secundum within 1 year of birth and when there is a potential lacuna formed at the fossa ovalis of the interatrial septum, which accounts for approximately 25% of adults (1). PFO is associated with a variety of diseases, including cryptogenic stroke, migraine, platypnea-orthodeoxia, decompression illness, and obstructive sleep apnea (2). Four randomized controlled trials have shown that transcatheter closure of PFO is superior to medical therapy alone in reducing the risk of effective stroke recurrence (3–6). Some studies also point to a higher benefit of direct PFO closure compared to antiplatelet therapy (7). Although X-ray fluoroscopy to see a guidewire crossing the interatrial septum is the most accurate method to confirm the presence of PFO, the above methods are invasive and cannot be widely carried out in clinical work (8).

At present, many studies have shown that TCD as first-choice screening tool for PFO and transesophageal echocardiography (TEE) examination is the gold standard for the diagnosis of PFO (9, 10), while an effective Valsalva maneuver is an accurate method for the diagnosis of the right-to-left shunt of PFO (11). To better observe the right-to-left shunt of PFO, an appropriate provocative maneuver needs to be performed. The Valsalva maneuver is currently the most commonly used provocative maneuver, but some patients cannot complete an effective Valsalva maneuver due to intubation and other reasons, which will lead to false negative results in the detection of a right-to-left shunt of PFO by TEE (12). Therefore, a simpler and more feasible maneuver is required to cooperate with TEE to detect PFO.

Central to the Valsalva maneuver is increased vena cava return, thereby increasing the right atrial pressure, so we explored other agonistic maneuvers that are similar to the principle of the Valsalva maneuver and that can be effectively completed in the TEE state. Muscle pumps play a large role in the human body, and each group of muscles can squeeze blood vessels through a contraction, thereby pumping blood back to the heart, and the calf muscle pumps are the most important (13). Calf muscle pump tensing (TENSE) is a novel provocative maneuver that uses the action of the calf muscle pumps. In this study, we compared and analyzed the Valsalva maneuver and Valsalva + TENSE combined maneuver, hoping to provide a simple and effective maneuver, which can compensate for the limitations of TEE in detecting right-to-left shunts while improving the detection rate of PFO.

## 2. Materials and methods

### 2.1. Patient population

Between September 2021 and March 2022, we investigated 171 patients who were highly suspected of having PFO after clinical and TTE (transthoracic echocardiography, TTE) examinations in Shaoxing People's Hospital, and five patients who were newly diagnosed with an atrial septal defect on TEE were excluded. Eventually, 166 patients were included in this study. Among them, there were 81 males and 85 females, aged 26 to 78 years, with an average of 53 ± 13 years, of which 82 (49%) had an unexplained stroke, 64 (39%) had migraine, 12 (7%) had a transient ischemic attack, and 8 (5%) had hypoxemia (Table 1). After ruling out any contraindications, TEE was performed. The exclusion criteria included: atrial fibrillation, patients with major heart disease including moderate and severe valvular disease, moderate and severe left ventricular systolic dysfunction, congenital heart disease and cardiac thrombosis, tumor, or vegetation. Before performing the examination, each patient was trained on the Valsalva maneuver and TENSE maneuver. All patients showed sinus rhythm during the examination. Before enrollment, all patients or their legal guardians signed informed consent forms. This study was approved by the ethics committee of the local hospital.

**Table 1:** Patient characteristics.

**Variables**	**Value**
Age (y)	53 ± 13
Sex (male)	81 (49%)
Hypertension, n (%)	45 (27%)
Diabetes mellitus, n (%)	9 (5%)
Coronary heart disease, n (%)	7 (4%)
Cryptogenic stroke, n (%)	82 (49%)
Migraine, n (%)	64 (39%)
Transient ischemic attack, n (%)	12 (7%)
Hypoxemia, n (%)	8 (5%)

Data are expressed as the mean ± SD or as the number (percentage) of patients.

Each patient underwent contrast transesophageal echocardiography. Each subject was subjected to comprehensive TEE evaluations. A Philips EPIQ 7C (Philips Ultrasound, Bothell, Washington, USA) was used with a transesophageal three-dimensional matrix probe X8-2t (frequency 2-8 MHz). Local anesthesia was administered into the oropharynx using 2% lidocaine gel. The saline contrast agent was produced by 1 mL of air, 1 mL of blood, and 8 mL of saline, which was shaken 80 times per minute between two 10 mL syringes connected to the 3-way stopcock and was rapidly injected into the antecubital vein (12). Bulging of the interatrial septum in the direction of the left atrium was used as a criterion for effective provocative maneuvering, and direct observation of PFO under TEE was used as a criterion for confirming the diagnosis of PFO (11). Grading was performed according to the number of microbubbles, and the number of microbubbles appearing in the left heart chamber on resting single-frame images was graded as follows: Grade 0 (zero microbubbles/frame); Grade 1 (mild, < 10 microbubbles/frame); Grade 2 (moderate, 11 to 30 microbubbles/frame); and Grade 3 (severe, > 30 microbubbles/frame) (14).

### 2.2. Definitions

#### 2.2.1. Valid provocative maneuver

**Figure 1:**
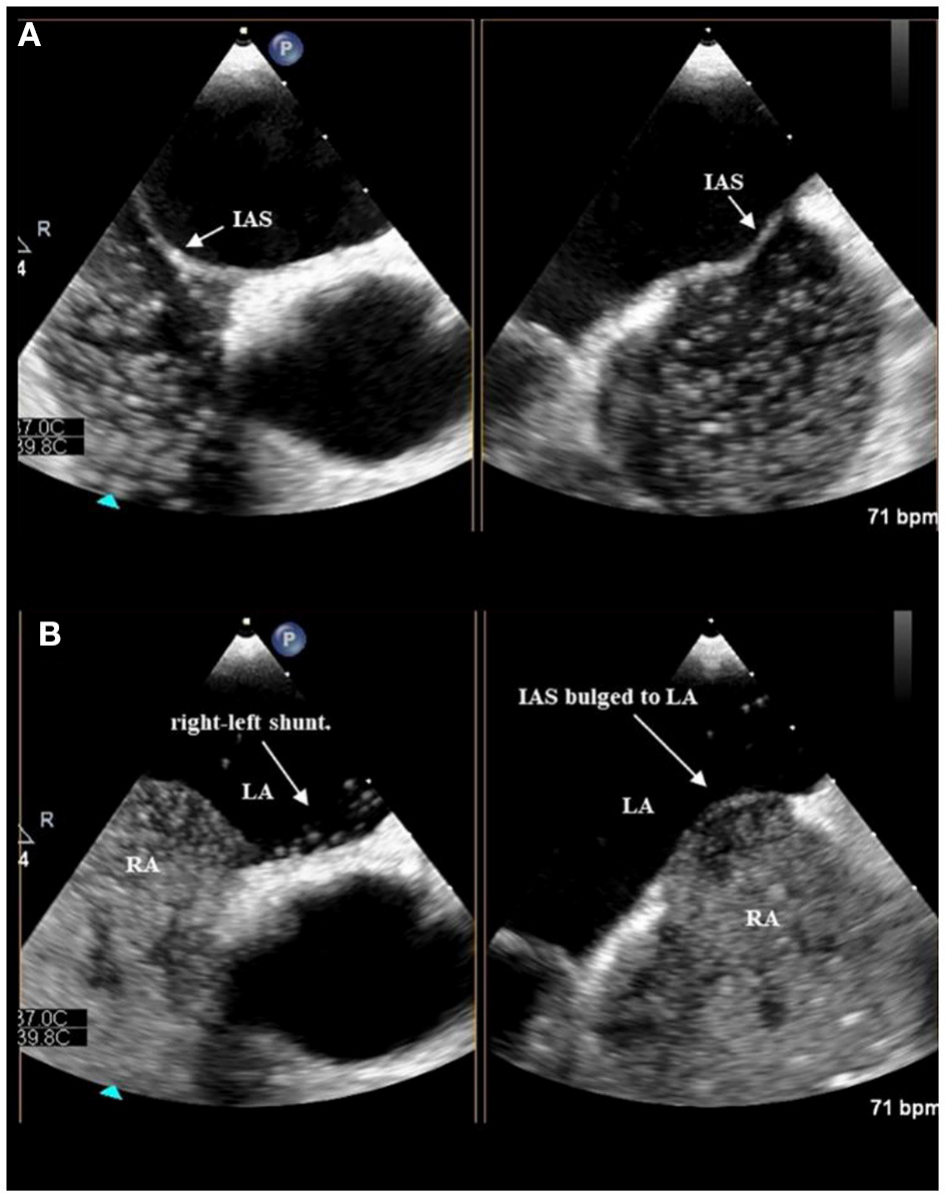
Position of interatrial septum at rest before TENSE **(A)**. The IAS bulged to LA after TENSE, At the same time, a bunch of microbubbles can be seen to overflow from the foramen ovale **(B)**.

The biatrial-bicaval view was selected as the best image for observing the interatrial septum by adjusting the angle, usually 90°-110°. An effective provocative maneuver is defined as atrial septal bulging to the left atrium after completion of the provocative maneuver, which is defined as atrial septal displacement to the left or bulging to the left and is qualitatively assessed by the visual judgment of the examiner (Figure 1).

#### 2.2.2. Standard Valsalva

A standard Valsalva maneuver refers to the patient inhaling normally or deeply, then rapidly closing the mouth and nose, exhaling forcefully against the closure for 15–20 s, and releasing exhalation when the microbubbles enter the right atrium, and the right atrium is then completely filled with microbubbles.

#### 2.2.3. Calf muscle pump tensing

**Figure 2:**
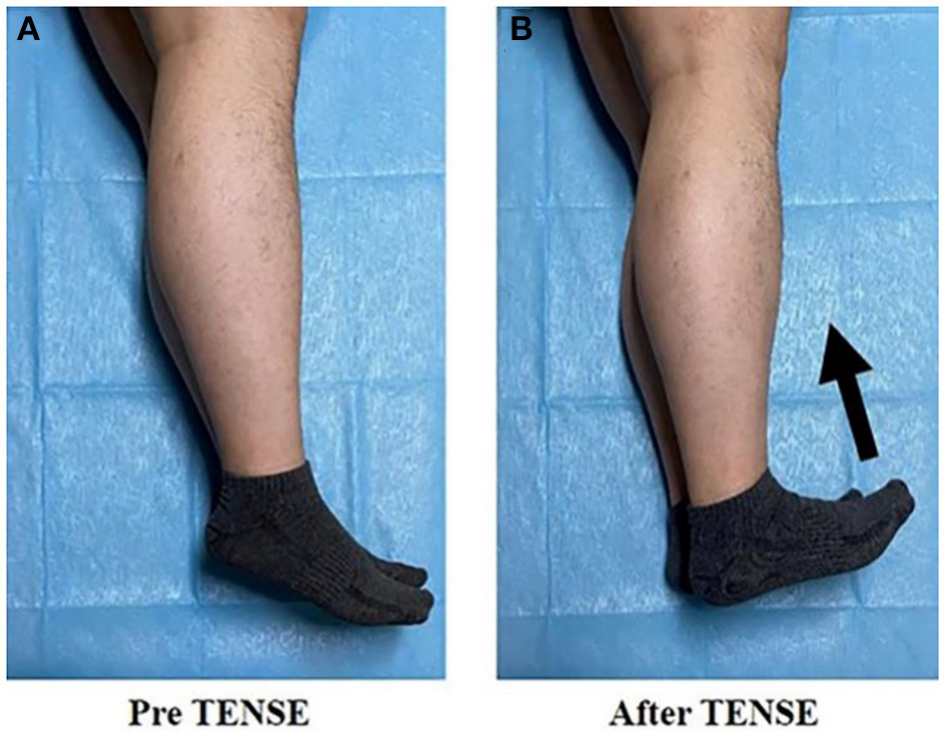
Pre TENSE: represents lower limb posture at rest **(A)**; after TENSE: represents TENSE motor posture **(B)**.

In the TENSE maneuver, the patients are first asked to keep both lower limbs straight. Before the right atrium is filled with microbubbles, the patient must immediately perform bilateral ankle dorsiflexion maneuvers with maximum strength and maintain the dorsiflexion for 3–5 s (Figure 2).

#### 2.2.4. Combined maneuver

Patients need to practice the combined maneuver of Valsalva and TENSE in advance, with the rapid expiratory moment of Valsalva action as the node (the third stage of Valsalva action), and immediately perform bilateral ankle dorsiflexion movements and maintain the dorsiflexion for 3–5 s.

### 2.3. Demographic data collection

Clinical data included age, sex, presence of hypertension, diabetes mellitus, unexplained stroke, migraine, transient ischemic attack, and hypoxemia.

### 2.4. Observer variability

The agreement between the TENSE maneuver, Valsalva maneuver, and Valsalva + TENSE combined maneuver was evaluated. A second observer blinded to the results independently evaluated whether the different provocative maneuvers were effective and whether there was a right-to-left shunt to assess the interobserver variability.

### 2.5. Statistical analysis

Continuous variables are expressed as the mean ± SD. Categorical variables are expressed as the number of patients (n) and percentage (%). The chi-square test was used to compare the effective provocative rate and PFO detection rates between the different provocative maneuvers.

The Wilcoxon matched pairs test was used to compare quantitative data for right-to-left shunting between the provocative maneuvers (with Bonferroni correction).

To assess the sensitivity of the different provocative maneuvers to detect PFO and while using any provocative maneuver that diagnosed PFO patients as the gold standard, the agreement between the different provocative maneuvers was tested using k statistics. A *P* < 0.05 was considered statistically significant. All statistical analyses were performed using IBM SPSS 26.0 software.

## 3. Results

### 3.1. Comparison between the provocative maneuvers

**Figure 3:**
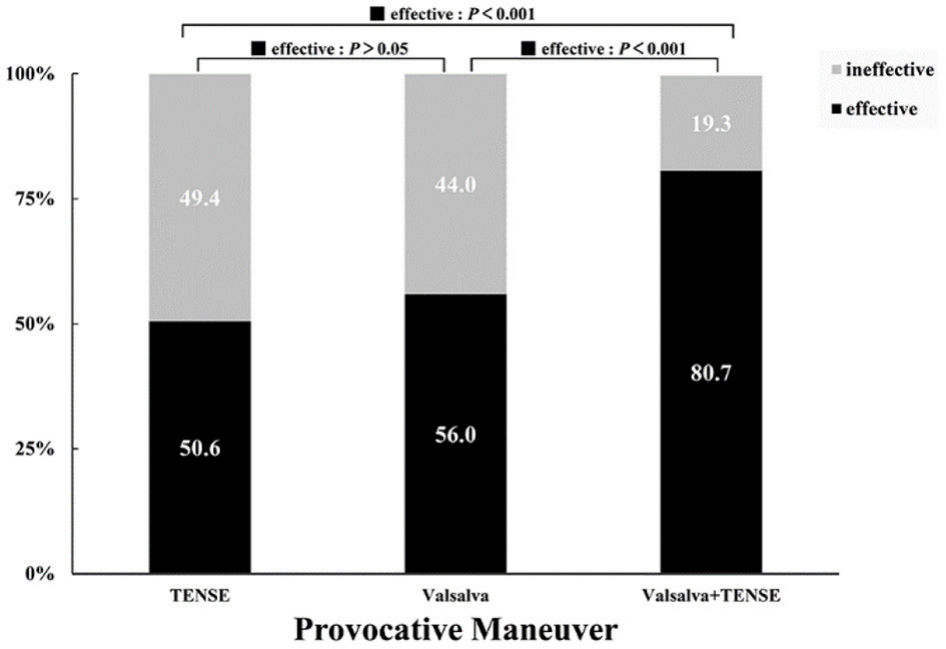
Comparison of the effective provocative rates of different provocative maneuvers.

All 166 patients with suspected PFO underwent the TENSE maneuver, Valsalva maneuver, and Valsalva + TENSE combined maneuver. The effective provocative rate of TENSE was 84 (50.6%), the effective provocative rate of Valsalva was 93 (56.0%), and the effective provocative rate of Valsalva + TENSE was 134 (80.7%). There was a significant difference in the overall means among the three groups (χ^2^ = 36.5, *P* < 0.001). There was no significant difference between the Valsalva maneuver and TENSE maneuver (*P* > 0.05), and there were significant differences between the Valsalva + TENSE maneuver and the Valsalva maneuver and TENSE maneuver (*P* < 0.001) (Figure 3). We performed stratified analysis according to baseline data gender, and the effective provocative rate of TENSE maneuver was higher in males than in females, which was statistically different (TENSE, male 51/81 [63.0%] vs. female 33/85 [38.8%], χ^2^= 9.67, *P* = 0.002).

### 3.2. PFO detection rate comparison

**Figure 4:**
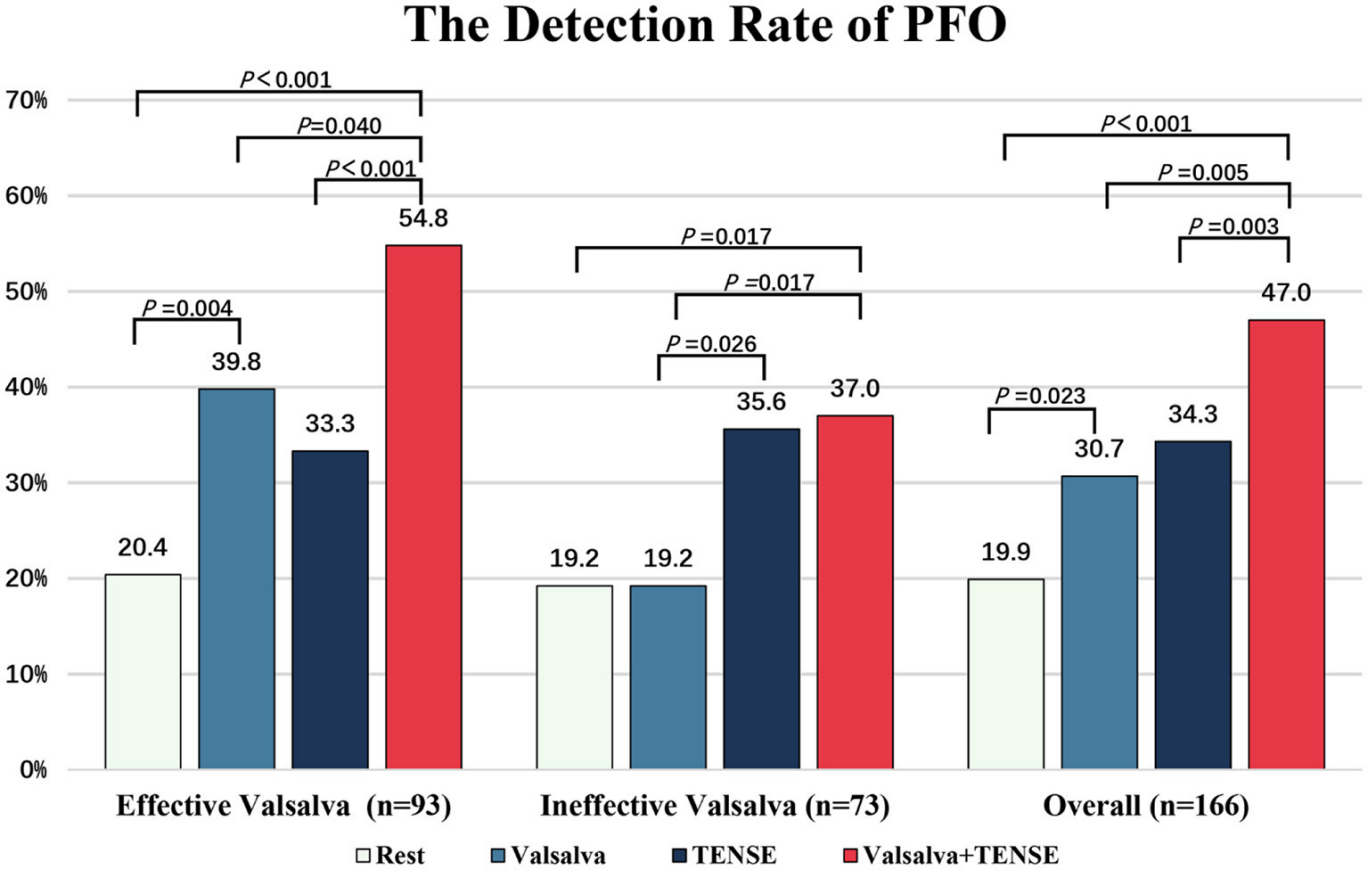
The detection rate of PFO after rest, Valsalva, TENSE, Valsalva + TENSE in different populations. The overall PFO detection rate (*n* = 166) and the rate in patients who could perform an effective (*n* = 93) or ineffective (*n* = 73) Valsalva maneuver.

**Figure 5:**
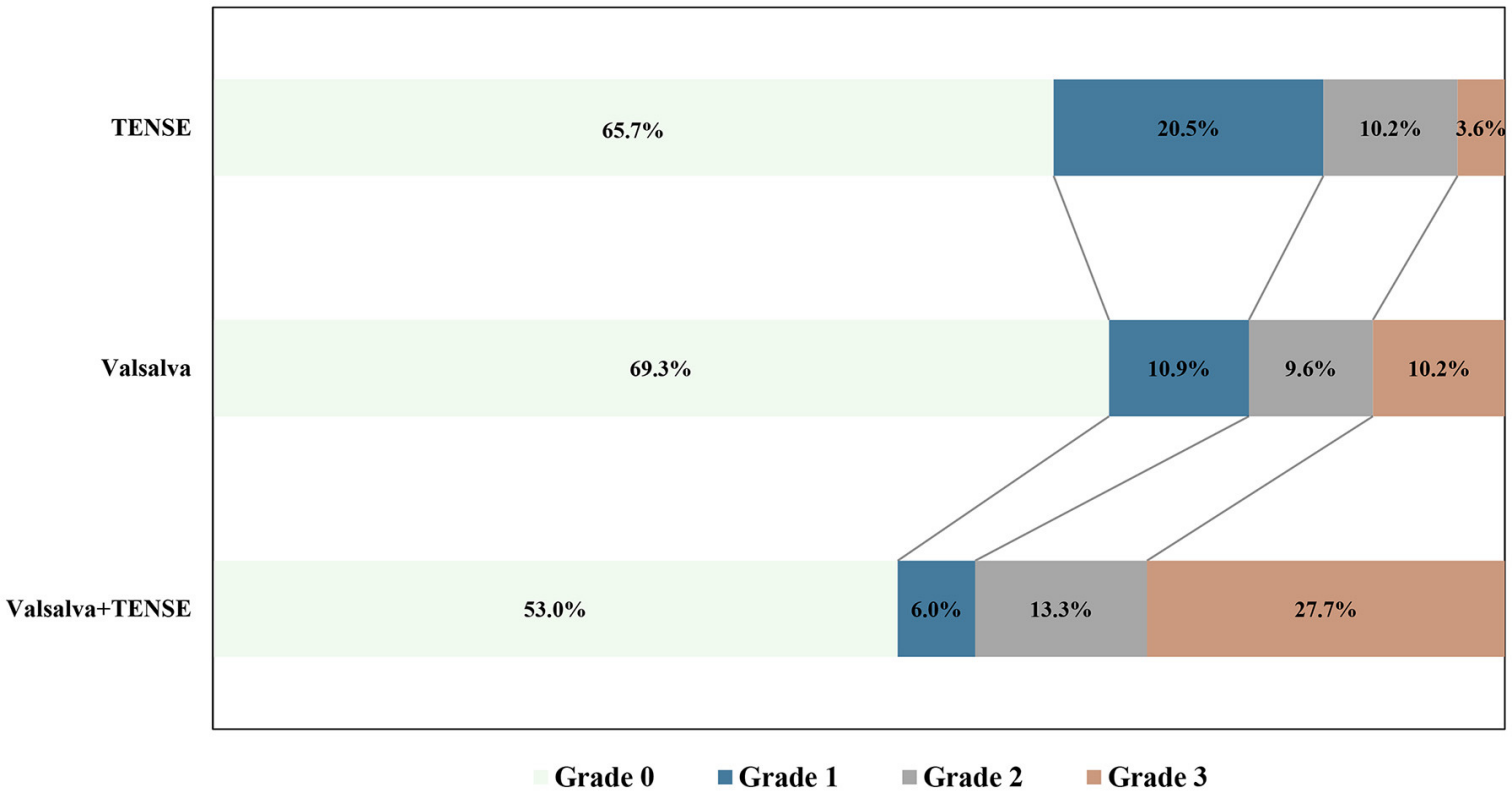
Comparison of the right-to-left shunt of PFO grade results after different provocative maneuvers.

Overall, the detection rate of PFO by the TENSE maneuver was not different from that of the Valsalva maneuver (Valsalva 51/166 [30.7%] vs. TENSE 57/166 [34.3%], *P* > 0.05), and the detection rate of PFO by the combined maneuver was higher than that by the Valsalva maneuver alone (Valsalva +TENSE 78/166 [50.1%] vs. Valsalva 51/166, [30.7%], *P* = 0.017). The patients were divided into the effective Valsalva group (*n* = 93) and ineffective Valsalva group (*n* = 73) according to whether they could perform effective Valsalva movements. In the effective Valsalva group, the provocative maneuver could improve the sensitivity of PFO detection (rest vs. provocative Maneuver, *P* < 0.001); although the Valsalva maneuver had a higher PFO detection rate, it was not significantly different from TENSE (Valsalva 37/93 [39.8%] vs. TENSE 31/93 [33.3%], *P* > 0.05), the combined maneuver had the highest PFO detection rate and was significantly different compared to the Valsalva maneuver(Valsalva +TENSE 51/93 [54.8%] vs. Valsalva 37/93 [39.8%], *P* = 0.040). In the ineffective Valsalva group, the combined maneuver showed a higher PFO detection rate (Valsalva + TENSE 27/73 [37.0%] vs. Valsalva 14/73 [19.2%], *P* < 0.001). The detection rate of PFO by TENSE was significantly higher than that by Valsalva (TENSE 26/73 [35.6%] vs. Valsalva14/73[19.2%], *P* < 0.001). The combined maneuver had a higher PFO detection rate than the rest and the Valsalva maneuver, and there was no significant difference compared with TENSE (*P* > 0.05) (Figure 4). By the multiple sample rank sum test, it was found that there was significant difference in the right-to-left shunt grade between the three activation tests, (χ^2^ = 23.1, *P* < 0.001). Further pairwise analysis showed that the Valsalva and TENSE right-to-left shunt grades were not different (*P* = 0.690), while the grades of Valsalva + TENSE were different from those of Valsalva and TENSE (*P* < 0.001) (Table 2, Figure 5).

**Table 2:** Comparisons between TENSE and other provincial maneuvers.

**Commonly used provocative maneuvers**	**PFO diagnostic sensitivity**	**Patient cooperation required**	**Difficulty completing effective action**	**Can be combined with Valsalva**	**TEE sectional image Stability**
Valsalva	—	Yes	Hard	—	Bad
Forced expiratory	—	Yes	Hard	—	Bad
Deep inspiration	—	Yes	Hard	—	Bad
Cough	—	Yes	Hard	No	Bad
Müller (modified)	—	Yes	Hard	Yes	Bad
Abdominal compression	Better than ineffective Valsalva	No	—	Yes	Bad
IVC compression	Better than ineffective Valsalva	No	—	Yes	Good
TENSE	Better than ineffective Valsalva	Yes	Easy	Yes	Good

## 4. Discussion

### 4.1. Hemodynamics of PFO

In PFO patients at rest, the pressure changes in the left and right atria change with the cardiac cycle, and, most of the time, the left ventricular pressure is ~3–5 mmHg higher than the right atrial pressure, which may lead to left-to-right shunting at the atrial level in PFO patients (15). During atrial systole, the left atrium contracts slightly later than the right atrium, so the left and right atrial pressure may reverse when the right atrium begins to contract and when the left atrium has not yet started to contract, which may produce a transient and a small right-to-left shunt (16). In some physiological or pathological conditions, the right atrial pressure may also be transient or persistently higher than the left atrial pressure. Physiological states, including coughing, sneezing, defecation, body bending, and other movements, can lead to a transient increase in intrathoracic pressure, increased abdominal pressure, and then instantaneous increased right atrial pressure. Pathological conditions, including acute pulmonary embolism, pulmonary hypertension, and massive tricuspid regurgitation, can further increase right atrial pressure (17). When microthrombi are present in the venous system or *in situ*, thrombosis of the foramen ovale may lead to emboli entering the left ventricular system through patent foramen ovale channels to form paradoxical embolism, causing the occurrence of patent foramen ovale-associated stroke (18, 19).

### 4.2. Mechanism of the Valsalva maneuver

**Figure 6:**
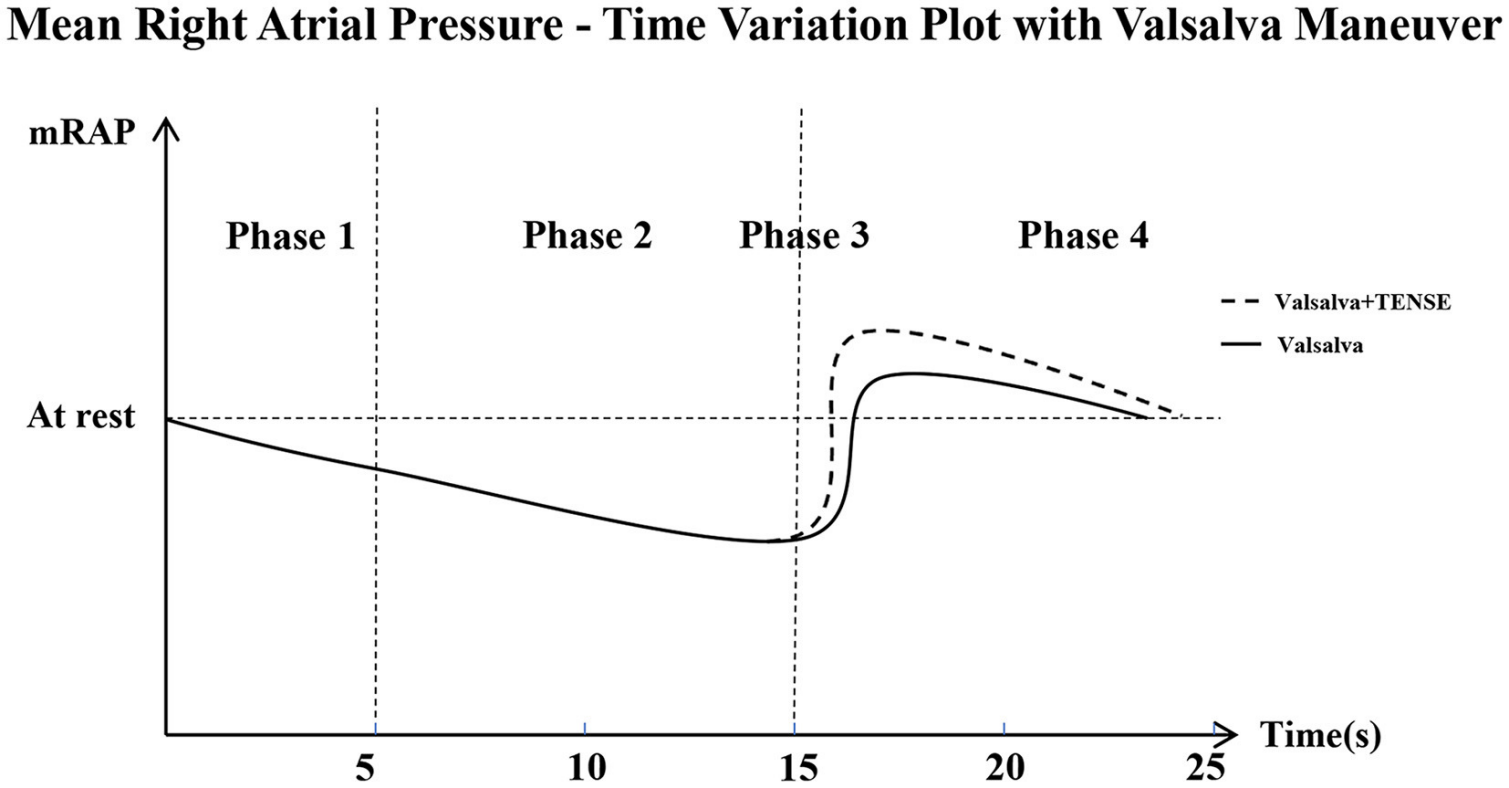
Mean Rigut Arital Pressure–Time Variation Plot with Valsalva maneuver, This is a schematic that shows how mean right atrial pressure changes over time. mRAP, Right Atrial Mean Pressure During a Cardiac Cycle. Phase 1 (0-5s); Phase 2 (5-15s); Phase 3 (15-20s); Phase 4 (20-25s). The Valsalva + TENSE combined maneuver increases mRAP more than the Valsalva maneuver in Phase 3.

The Valsalva maneuver is the most commonly used provocative maneuver. Its main mechanism of action is to reduce the left atrial pressure and increase the right atrial pressure, which causes the reversal of the left atrial and right atrial pressure gradient and causes right-to-left shunting of PFO. According to the characteristic FPO hemodynamics, it can be divided into four phases. Phase 1: During the first few seconds, the left and right atrial and vena cava walls are compressed due to increased intrathoracic pressure, causing a decrease in return blood flow; Phase 2: within 5–15 s, the intrathoracic pressure continues to rise; the mean right atrial pressure continues to decrease. However, the left ventricular preload decreases, the cardiac output decreases, and at this time, the right atrial pressure may be slightly higher than the left atrial pressure. Phase 3: It begins with the cessation of the Valsalva maneuver, the intrathoracic pressure rapidly decreases, resulting in increased instantaneous reflux of the vena cava and causing a significant increase in the mean right atrial pressure when the maximum gradient between right atrial and left atrial pressure is formed. Phase 4: The vena cava reflux gradually resolves, and the mean right atrial pressure returns to the resting state (Figure 6).

### 4.3. Mechanisms of PFO detection by the calf muscle pump tensing maneuver

The results of this study showed that the PFO detection rate of TENSE was higher than that of the resting state and similar to that of the Valsalva maneuver. The core of the TENSE maneuver is instantaneous dorsiflexion of the ankle joint, which activates both the lower limb muscle pump and the venous pump (13, 20). The pressure generated by the calf muscle pumps during each contraction is more than 200 mmHg, and the blood volume that can be discharged is ~30–40 mL, which is called “the surrounding heart” (21). The calf muscle pump includes the soleus pump and gastrocnemius pump, and the ankle dorsiflexion action is completed by the contraction of the tibialis anterior, extensor digitorum longus extensor pollicis longus, and peroneus tertius muscles (13). Ankle dorsiflexion movements have a direct effect on the soleus and gastrocnemius muscles, which are elongated and contracted as antagonistic muscles. Also, the dorsiflexion agonistic and antagonistic muscles produce interaction forces, so the veins are compressed, thereby pumping the blood back to the heart. On the other hand, the venous pump constitutes the venous anti-gravity reflux system and pulse suction pump in the human body. During contraction, the muscle pressure that changes instantaneously due to dorsiflexion movements is activated, and the blood is further pumped back to the heart in a pulsed manner (22, 23). The combined action of the venous-muscle pump causes a significant increase in the instantaneous velocity of the inferior vena cava, and the venous return velocity is the main factor affecting the venous return volume. In most patients, the amount of venous return volume is the main factor determining the magnitude of cardiac preload. TENSE can significantly increase the return flow of the inferior vena cava and thus change the right atrial pressure, resulting in a larger instantaneous right atrial-left atrial pressure difference and ultimately causing a right-to-left shunt in PFO (Figure 6).

### 4.4. Comparisons between TENSE and Valsalva maneuver

The Valsalva maneuver has long been considered the most appropriate provocative maneuver for PFO detection by TEE (24). Hemodynamic studies have shown that an effective Valsalva maneuver can cause a significant increase in the left and right atrial gradients and has two major advantages: a longer time for the right atrial pressure to be greater than the left atrial pressure (25). At the end of Valsalva maneuver, the resistance of venous return is relieved, the right atrial pressure and pulmonary capillary pressure can be significantly increased, and the left atrial return bleeding mainly comes from the lung. However, the right atrial return bleeding comes from the superior and inferior vena cava because the blood volume stored in the lung is much less than the blood volume stored in the superior and inferior vena cava; therefore, the blood flow to the right atrium is much greater than that in the left atrium, eventually resulting in an instantaneous right atrial pressure that is significantly higher than the left atrial pressure. This causes the blood flow through the foramen ovale to shunt to the left atrium. Therefore, the detection rate of PFO and right-to-left shunt degree are influenced by the quality of the Valsalva maneuver. Studies have shown that approximately 60% of patients are unable to perform effective Valsalva maneuvers during TEE (26). By stimulating the esophagus with the probe, the patients may experience discomfort, which will decrease the effect of the Valsalva maneuver. the research of Devuyst et al. found that the number of microbubbles increases with expiratory pressure independent of strain duration (27). On the other side, end-expiration may cause mediastinal displacement due to intrathoracic pressure changes, resulting in the PFO not being in the image display area, which affects the direct observation of PFO. In this study, the effective provocative rate of the TENSE maneuver was higher than that of the Valsalva maneuver, but there was no significant difference between the two. The patient can easily complete the TENSE maneuver under intubation. The veins of both lower limbs are squeezed due to the stress effect generated by the calf muscle pump, which will produce a high value of instantaneous flow velocity, increase the instantaneous pressure of the left atrium, and finally cause the right atrial pressure to be greater than the left atrial pressure, achieving an effect similar to that of the Valsalva maneuver. It can reduce the false negative results caused by the insufficient increase in right atrial pressure due to stimulation in some patients. On the other hand, TENSE has no direct effect on the intrathoracic pressure, so the images are more stable than images taken during the Valsalva maneuver during TEE examination.

### 4.5. Comparisons between TENSE and other provocative maneuvers

Various studies have shown that other provocative maneuvers, such as abdominal compression, inferior vena cava compression, cough, deep inspiration, and the Müller maneuver, have good results as new provocative maneuvers (28) (Table 2). At present, the Valsalva maneuver and cough are commonly used in clinical practice, but it can be difficult for patients to cooperate during TEE examination, resulting in difficulty in reaching the standard. Abdominal compression and inferior vena cava compression can cause a right-to-left shunt by forming a vena cava blood flow block in a short time. They can not only cause the right atrial pressure to be higher than the left atrial pressure but also can increase the instantaneous impact force of the inferior vena cava blood on the foramen ovale, which is conducive to the detection of PFO. Both methods have better sensitivity for PFO diagnosis than the null Valsalva maneuver but cannot be effectively performed in patients with abdominal pain, percutaneous catheter intervention, and abdominal surgery, and it has little effect in obese patients (29, 30). The Müller maneuver requires a closed glottis and rapid forceful nasal inspiration, but the modified Müller maneuver does not require a closed glottis, but it does require rapid forceful nasal inspiration (31). The modified Müller maneuver and dynamic bed tilt can be used as auxiliary provocation maneuvers in TTE with the Valsalva maneuver. However, no trials have been performed in TEE examination, and its feasibility and effectiveness need to be further studied. For cough and cephalad tilt movement, the increase in the right atrial pressure also causes an increase in the pulmonary capillary wedge pressure, that is, the left atrial pressure will also increase (32). This causes a failure to achieve an effective right atrium-left atrial gradient. Other studies have shown that the use of rapid infusion, sublingual nitroglycerin, and femoral vein injection may improve the detection rate of PFO (33), and these methods may cause some adverse reactions and cannot be verified and carried out on a large scale in clinical practice. This study showed that TENSE is simple, and the patients can easily cooperate with the movement. TENSE can be used independently for PFO examination and can increase the detection rate of PFO, and TENSE can be used as an effective Valsalva auxiliary means to significantly improve the PFO detection rate in patients who cannot complete an effective Valsalva maneuver. TENSE is the “last kilometer” of the Valsalva maneuver.

### 4.6. Influences on the effectiveness of the calf muscle pump tensing maneuver

The core of the TENSE maneuver is to tension the calf muscle pumps through the ankle dorsiflexion maneuver so that the interaction force between the muscles and the veins is generated; the veins are squeezed by the muscle stress; and the overall direction of the stress is upward, which is the same as the direction of venous return to the heart, while activating the venous pump to increase the venous pressure, resulting in increased right atrial pressure. Although this movement, whether passive or active, will result in the mean and peak blood flow velocities in the common femoral vein to exceed the established resting levels, studies have shown that active motion produces higher peak and mean blood flow velocities than passive motion (34). Active motion produced a mean increase of 38% in the velocity change and 58% in the peak velocity, which were significantly greater than that produced by passive motion, while passive flexion and passive rotation produced only a 9% increase in the mean velocity (35). Therefore, it is particularly important to complete active and complete TENSE maneuvers, and TENSE maneuvers may not play a significant role in patients who develop acute or early recovery from cerebrovascular accidents. Studies have shown that varus foot is easily induced during the recovery of hemiplegic lower limb function after stroke, and this may be accompanied by different degrees of decrease in the lower limb muscle strength, which will also affect the effect of TENSE movement. On the other hand, patients with tetraplegia caused by acute myelitis and Guillain–Barré syndrome are also unable to complete effective TENSE movements. In this study, the TENSE maneuver formed an effective provocation in a higher proportion of male patients than in females, which may be because the muscles of males are generally more developed than those of females.

### 4.7. Clinical implications and contraindications of TENSE

Unlike the Valsalva maneuver, the TENSE maneuver can avoid the impact of stimulation, such as intubation, on patients and does not require suffocation. As mentioned earlier, patients who cannot perform the effective Valsalva maneuver for some reasons can perform the TENSE maneuver instead, and we found that the combined maneuver of Valsalva + TENSE had a higher right-to-left shunt PFO detection rate (47%) in 166 patients. Although no significant complications were found during our study, TENSE caused sudden compression of the lower limb veins, resulting in a sudden increase in the venous pressure, and some patients at high risk of VTE or in patients with lower limb venous thrombosis should avoid performing TENSE maneuvers to prevent fatal pulmonary embolism after thrombus detachment.

## 5. Limitations

Although the conclusions of this study are novel and clinically meaningful, there are also some limitations, and this study was performed in a single center and needs to be further validated by a large-scale multicenter study. Second, there was selection bias because the subjects in this study were patients with a high clinical suspicion of PFO. Third, this study only discussed the active TENSE test, and the patients did not perform the passive TENSE (assisting patients to perform foot dorsiflexion maneuvers) test, which needs further investigation in the future. Fourth, this study did not compare these maneuvers with other provocative maneuvers, such as abdominal compression and inferior vena cava compression. Finally, the calf muscle pump is one of the four groups of muscle pumps in the lower limb, and no comparative studies have been conducted for several other groups of muscle pumps, which need to be further explored in future studies.

## 6. Conclusions

In TEE examination, TENSE is a simple and effective provocative maneuver that is not inferior to the Valsalva maneuver. In particular, TENSE can be a good alternative to the Valsalva maneuver for patients who are unable to perform an effective Valsalva maneuver.

## Data availability statement

The raw data supporting the conclusions of this article will be made available by the authors, without undue reservation.

## Ethics statement

The studies involving human participants were reviewed and approved by Academic Ethics Committee of Shaoxing People's Hospital. The patients/participants provided their written informed consent to participate in this study. Written informed consent was obtained from the individual(s) for the publication of any potentially identifiable images or data included in this article.

## Author contributions

JZ, AC, and XL contributed conception and design of the study. JZ drafted the original article. The paper was revised by XL and ZJ. LZ, YH, and HS collected and organized the database. YT and YL performed the statistical analysis. All authors substantially contributed to the acquisition, analysis or interpretation of data, and approved the final version of the manuscript.

## References

[1] Mac Grory B Ohman EM Feng W Xian Y Yaghi S Kamel H. Advances in the management of cardioembolic stroke associated with patent foramen ovale. BMJ. (2022) 376:e063161. 10.1136/bmj-2020-063161 35140114

[2] Homma S Messe SR Rundek T Sun YP Franke J Davidson K. Patent foramen ovale. Nat Rev Dis Primers. (2016) 2:15086. 10.1038/nrdp.2015.86 27188965

[3] Lee PH Song JK Kim JS Heo R Lee S Kim DH. Cryptogenic stroke and high-risk patent foramen ovale: the defense-Pfo trial. J Am Coll Cardiol. (2018) 71:2335–42. 10.1016/j.jacc.2018.02.046 29544871

[4] Sondergaard L Kasner SE Rhodes JF Andersen G Iversen HK Nielsen-Kudsk JE. Patent foramen ovale closure or antiplatelet therapy for cryptogenic stroke. N Engl J Med. (2017) 377:1033–42. 10.1056/NEJMoa1707404 28902580

[5] Saver JL Carroll JD Thaler DE Smalling RW MacDonald LA Marks DS. Long-term outcomes of patent foramen ovale closure or medical therapy after stroke. N Engl J Med. (2017) 377:1022–32. 10.1056/NEJMoa1610057 28902590

[6] Mas JL Derumeaux G Guillon B Massardier E Hosseini H Mechtouff L. Patent foramen ovale closure or anticoagulation vs. antiplatelets after stroke N Engl J Med. (2017) 377:1011–21. 10.1056/NEJMoa1705915 28902593

[7] Wu Z Zhang C Liu N Xie W Yang J Guo H. A nomogram for predicting patent foramen ovale-related stroke recurrence. Front Neurol. (2022) 13:903789. 10.3389/fneur.2022.903789 35756923 PMC9218274

[8] Campbell BCV Khatri P. Stroke. Lancet (London, England). (2020) 396:129–42. 10.1016/S0140-6736(20)31179-X 32653056

[9] Pearson AC Labovitz AJ Tatineni S Gomez CR. Superiority of transesophageal echocardiography in detecting cardiac source of embolism in patients with cerebral ischemia of uncertain etiology. J Am Coll Cardiol. (1991) 17:66–72. 10.1016/0735-1097(91)90705-E 1987242

[10] Koutroulou I Tsivgoulis G Tsalikakis D Karacostas D Grigoriadis N Karapanayiotides T. Epidemiology of patent foramen ovale in general population and in stroke patients: a narrative review. Front Neurol. (2020) 11:281. 10.3389/fneur.2020.00281 32411074 PMC7198765

[11] Bernard S Churchill TW Namasivayam M Bertrand PB. Agitated saline contrast echocardiography in the identification of intra- and extracardiac shunts: connecting the dots. J Am Soc Echocardiogr. (2020). 10.1016/j.echo.2020.09.013 34756394

[12] Silvestry FE Cohen MS Armsby LB Burkule NJ Fleishman CE Hijazi ZM. Guidelines for the echocardiographic assessment of atrial septal defect and patent foramen ovale: from the american society of echocardiography and society for cardiac angiography and interventions. J Am Soc Echocardiogr. (2015) 28:910–58. 10.1016/j.echo.2015.05.015 26239900

[13] Uhl JF Gillot C. Anatomy of the veno-muscular pumps of the lower limb. Phlebology. (2015) 30:180–93. 10.1177/0268355513517686 24415543

[14] Hahn RT Abraham T Adams MS Bruce CJ Glas KE Lang RM. Guidelines for performing a comprehensive transesophageal echocardiographic examination: recommendations from the american society of echocardiography and the society of cardiovascular anesthesiologists. J Am Soc Echocardiogr. (2013) 26:921–64. 10.1016/j.echo.2013.07.009 23998692

[15] Wright SP Dawkins TG Eves ND Shave R Tedford RJ Mak S. Hemodynamic function of the right ventricular-pulmonary vascular-left atrial unit: normal responses to exercise in healthy adults. Am J Physiol Heart Circ. (2021) 320:H923–H41. 10.1152/ajpheart.00720.2020 33356960

[16] Sun JP Stewart WJ Hanna J Thomas JD. Diagnosis of patent foramen ovale by contrast versus color doppler by transesophageal echocardiography: relation to atrial size. Am Heart J. (1996) 131:239–44. 10.1016/S0002-8703(96)90347-6 8579014

[17] Thibodeau JT Jenny BE Maduka JO Divanji PH Ayers CR Araj F. Bendopnea and risk of adverse clinical outcomes in ambulatory patients with systolic heart failure. Am Heart J. (2017) 183:102–7. 10.1016/j.ahj.2016.09.011 27979033

[18] Lechat P Mas JL Lascault G Loron P Theard M Klimczac M. Prevalence of patent foramen ovale in patients with stroke. N Engl J Med. (1988) 318:1148–52. 10.1056/NEJM198805053181802 3362165

[19] Samuel S Reddy ST Parsha KN Nguyen T Reddy Indupuru HK Sharrief AZ. Routine surveillance of pelvic and lower extremity deep vein thrombosis in stroke patients with patent foramen ovale. J Thromb Thrombolysis. (2021) 51:1150–6. 10.1007/s11239-020-02262-w 32888135

[20] Pi H Ku H Zhao T Wang J Fu Y. Influence of ankle active dorsiflexion movement guided by inspiration on the venous return from the lower limbs: a prospective study. JNR. (2018) 26:123–9. 10.1097/jnr.0000000000000214 28858972

[21] Kropp AT Meiss AL Guthoff AE Vettorazzi E Guth S Bamberger CM. The efficacy of forceful ankle and toe exercises to increase venous return: a comprehensive doppler ultrasound study. Phlebology. (2018) 33:330–7. 10.1177/0268355517706042 28478746

[22] Kirby BS Carlson RE Markwald RR Voyles WF Dinenno FA. Mechanical influences on skeletal muscle vascular tone in humans: insight into contraction-induced rapid vasodilatation. J Physiol. (2007) 583:861–74. 10.1113/jphysiol.2007.131250 17495044 PMC2277182

[23] Lurie F Kistner RL Eklof B Kessler D. Mechanism of venous valve closure and role of the valve in circulation: a new concept. J Vasc Surg. (2003) 38:955–61. 10.1016/S0741-5214(03)00711-0 14603200

[24] Pstras L Thomaseth K Waniewski J Balzani I Bellavere F. The valsalva manoeuvre: physiology and clinical examples. Acta Physiol (Oxf). (2016) 217:103–19. 10.1111/apha.12639 26662857

[25] Pfleger S Konstantin Haase K Stark S Latsch A Simonis B Scherhag A. Haemodynamic quantification of different provocation manoeuvres by simultaneous measurement of right and left atrial pressure: implications for the echocardiographic detection of persistent foramen ovale. Eur J Echocardiogr. (2001) 2:88–93. 10.1053/euje.2000.0052 11882433

[26] Rodrigues AC Picard MH Carbone A Arruda AL Flores T Klohn J. Importance of adequately performed valsalva maneuver to detect patent foramen ovale during transesophageal echocardiography. J Am Soc Echocardiogr. (2013) 26:1337–43. 10.1016/j.echo.2013.07.016 23993693

[27] Devuyst G Piechowski-Józwiak B Karapanayiotides T Fitting JW Kémeny V Hirt L. Controlled contrast transcranial doppler and arterial blood gas analysis to quantify shunt through patent foramen ovale. Stroke. (2004) 35:859–63. 10.1161/01.STR.0000119384.28376.EB 14988580

[28] Thiagaraj AK Hughes-Doichev R Biederman RWW. Provocative maneuvers to improve patent foramen ovale detection: a brief review of the literature. Echocardiography. (2019) 36:783–6. 10.1111/echo.14297 30803022

[29] Takaya Y Watanabe N Ikeda M Akagi T Nakayama R Nakagawa K. Importance of abdominal compression valsalva maneuver and microbubble grading in contrast transthoracic echocardiography for detecting patent foramen ovale. J Am Soc Echocardiogr. (2020) 33:201–6. 10.1016/j.echo.2019.09.018 31837927

[30] Yamashita E Murata T Goto E Fujiwara T Sasaki T Minami K. Inferior vena cava compression as a novel maneuver to detect patent foramen ovale: a transesophageal echocardiographic study. J Am Soc Echocardiogr. (2017) 30:292–9. 10.1016/j.echo.2016.11.011 28024853

[31] Becnel M Kerut EK Nanda NC. Peripheral saline contrast with valsalva and a modified müller's maneuver may improve detection of atrial level right-to-left shunts: a preliminary observation. Echocardiography. (2019) 36:651–3. 10.1111/echo.14244 30592781

[32] Gorgulu S Eksik A Eren M Celik S Uslu N Yildirim A. Assessment of the effects of various maneuvers on both atrial pressure changes. Int J Cardiol. (2003) 92:241–5. 10.1016/S0167-5273(03)00096-2 14659859

[33] Johansson MC Helgason H Dellborg M Eriksson P. Sensitivity for detection of patent foramen ovale increased with increasing number of contrast injections: a descriptive study with contrast transesophageal echocardiography. J Am Soc Echocardiogr. (2008) 21:419–24. 10.1016/j.echo.2007.08.030 17928199

[34] Shimizu Y Kamada H Sakane M Aikawa S Mutsuzaki H Tanaka K. A novel exercise device for venous thromboembolism prophylaxis improves venous flow in bed versus ankle movement exercises in healthy volunteers. J Orthop Surg (Hong Kong). (2017) 25:2309499017739477. 10.1177/2309499017739477 29137566

[35] Sochart DH Hardinge K. The relationship of foot and ankle movements to venous return in the lower limb. J Bone Joint Surg Br. (1999) 81:700–4. 10.1302/0301-620X.81B4.0810700 10463749

